# Ossifying fasciitis of the proximal lower extremity

**DOI:** 10.1080/23320885.2024.2309970

**Published:** 2024-02-05

**Authors:** Sydney Bormann, Tiffany Bender, Nicholas Olson, Jason Fowler

**Affiliations:** a Sanford School of Medicine, University of South Dakota, Sioux Falls, SD, USA; b Physicians Laboratory, Ltd, Sioux Falls, SD, USA; c Avera Medical Group Plastic and Reconstructive Surgery, Sioux Falls, SD, USA

**Keywords:** Ossifying fasciitis, soft tissue tumor

## Abstract

Ossifying fasciitis is a rare benign tumor of heterotopic bone formation within fascial tissue. We present a case of a 23-year-old female with a nontraumatic painful mass of the left proximal thigh identified as ossifying fasciitis, a lesion that must be considered in the differential diagnosis of soft tissue tumors.

## Introduction

Ossifying fasciitis is a benign soft tissue tumor that arises within fascial tissue and demonstrates heterotopic ossification [1,2]. Ossifying fasciitis is classified as a subtype of nodular fasciitis which has two other subtypes: subcutaneous and muscular. Although nodular fasciitis is a relatively common lesion that mimics malignant soft tissue tumors, ossifying fasciitis is a rare variant [3]. Ossifying fasciitis most commonly occurs between the ages of 20 to 30 years old [4]. These lesions have an affinity for fascia of the upper extremities, chest, back, head, and neck as well as tissue adjacent to joints, including tendons [1,3,5].

Radiographic findings of ossifying fasciitis have not been well described in literature but may show soft tissue swelling or calcifications depending on the stage of development [6,7]. Histological examination is often diagnostic, as ossifying fasciitis typically has features of both nodular fasciitis and myositis ossificans. In addition to classic areas of nodular fasciitis that demonstrate irregularly arranged proliferating myofibroblastic spindle or stellate cells loosely arranged in a myxoid matrix, ossifying fasciitis has metaplastic bone and can be less circumscribed than conventional nodular fasciitis [8]. Additionally, unlike myositis ossificans, the metaplastic bone of ossifying fasciitis lacks zonal maturation allowing distinction between the two entities [3].

Treatment of ossifying fasciitis varies, as there are no evidence-based guidelines for managing these lesions. Existing literature describes forms of nodular fasciitis treated with modalities ranging from spontaneous regression to surgical excision [3,8].

## Case report

**Figure 1.**
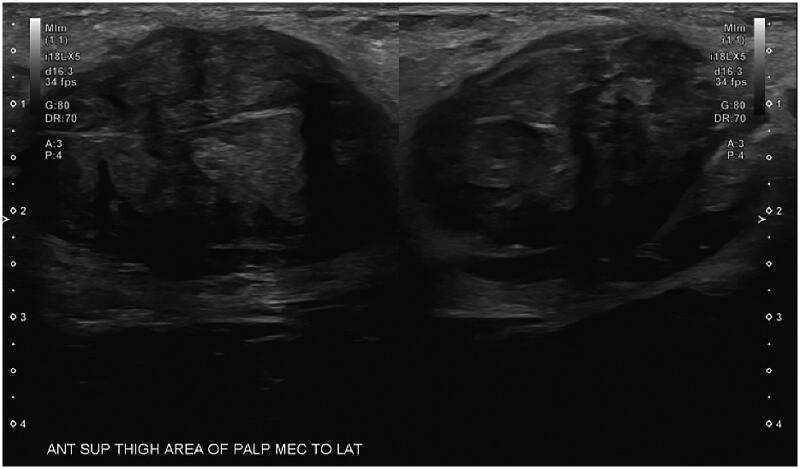
Ultrasound of the left upper thigh demonstrating a hypoechoic mass measuring 3.5 x 3 x 2.4 cm.

A 23-year-old female presented with a two-month history of a painful, palpable, slowly growing mass at the junction of her left vulvar crease and proximal thigh, which caused pain with sitting and movement. There was no associated erythema or warmth. There was no history of trauma to the area and hematology labs were unremarkable. Prior to presenting to plastic surgery clinic, the patient was evaluated by her primary care provider (PCP) who ordered an ultrasound with doppler which demonstrated a hypoechoic mass measuring 3.5 × 3 x 2.4 cm in the left inguinal region with hilar blood flow. Based on the anatomical location, the ultrasound findings were suspicious for lymphadenopathy (Figure 1) and further evaluation with a CT or biopsy was recommended.

**Figure 2.**
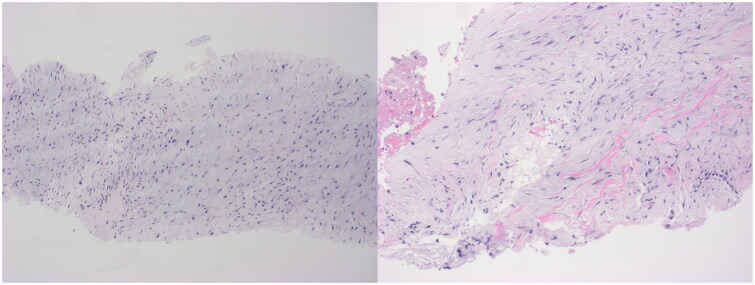
Histological examination showed haphazardly arranged spindle to stellate cells arranged in a myxoid matrix with mucin pools. FISH analysis revealed the presence of a USP6 rearrangement.

One week after the initial ultrasound, an ultrasound-guided biopsy was performed (Figure 2) which showed irregularly arranged bland spindle cells with myxoid stroma. FISH analysis was performed and was positive for rearrangement of the USP6 (17p13.2) locus and negative for fusion of the MYH9 (22q12.3) and USP6 (17p13.2) loci, supporting a diagnosis of nodular fasciitis.

**Figure 3.**
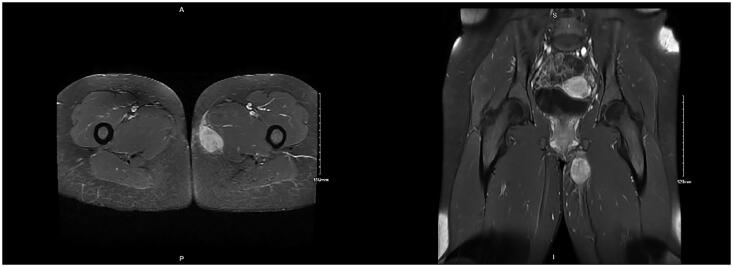
MRI demonstrating soft tissue mass measuring 4.8 x 2.7 x 4.0 cm extending into the left gracilis muscle.

A CT was performed two weeks after the biopsy was collected. The mass was not visualized on CT, and MRI was performed one month following CT. MRI revealed a mass measuring 4.8 × 2.7 × 4.0 cm extending from the uppermost portion of the left gracilis muscle to the inferior left pubic ramus, which resulted in mass effect on the gracilis muscle (Figure 3). The mass appeared heterogeneous but primarily isointense compared to muscle on T1, hyperintense on T2, and diffusely enhancing after contrast was administered. There was a small tail of enhancing tissue just anterior to the dominant mass. No enlarged left inguinal lymph nodes were found, and no surrounding edema was appreciated.

**Figure 4.**
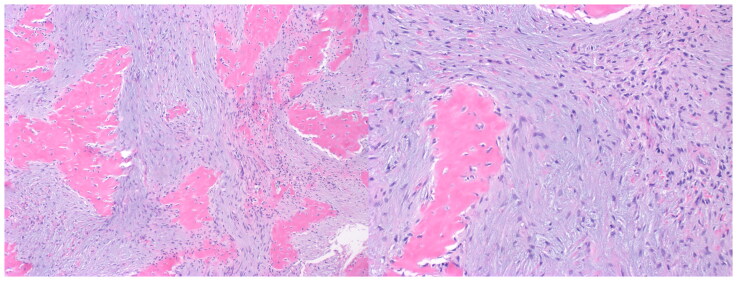
The resection specimen showed similar spindle to stellate cells, and also showed irregular fragments of osteoid, consistent with ossifying fasciitis.

Surgery was performed three months after initial presentation and two months after presentation to plastic surgery clinic. Dissection revealed a poorly encapsulated mass intricately associated with the gracilis muscle fascia extending to the pubic ramus. On macroscopic examination, the specimen measured 6.5 × 4 x 2.5 cm in size and appeared gray-white in color. Its structure was solid, focally gritty and calcified, rubbery, and indurated. Histological analysis (Figure 4) revealed short intersecting fascicles of cytologically uniform myofibroblastic spindle cells having ovoid or tapering nuclei and palely eosinophilic cytoplasm. However, the specimen also demonstrated multiple foci of irregular fragments of metaplastic bone. Given the molecular analysis from the biopsy specimen showing a USP6 rearrangement and the osteoid formation, a diagnosis of ossifying fasciitis was rendered.

At the 4-month postoperative follow-up visit, the patient had no complications and no recurrence. No residual edema or mass was present. The patient reported complete resolution of pain and symptoms.

## Discussion

Ossifying fasciitis, a variant of nodular fasciitis, is a rapidly growing soft tissue mass that occurs through a process of abnormal extraskeletal ossification [9]. This entity occurs after soft tissue trauma in 10-15% of cases; however, it can also occur after surgery or without any predisposing factors [4,9]. Growth of the mass is typically rapid with an early phase of hyperplasia of soft tissue followed by formation of calcification and bone tissue. A well-circumscribed mass forms within one to two months and causes pain and local inflammation [3]. Inflammation usually resolves, but a calcified soft tissue mass may persist. This condition is often managed with anti-inflammatory medication and direct local excision if symptomatic [8,10]. No cases of malignant transformation have been described [3].

While the pathophysiology of ossifying fasciitis is not well understood, in the acquired type, it has been noted to develop after trauma to the affected tissue, such as blunt force trauma, fractures of adjacent bone, joint replacements, or nerve injury. One study suggests macrophages responding to injured tissue secrete osteogenic factors and induce local stem cells and/or progenitor cells, which may trigger the development of tissue ossification [5]. This method of adaptive immune response seems to have no role in the genetic etiology of ossification, however [5]. Rearrangement of the ubiquitin-specific protease 6 (USP6) gene is seen in nodular fasciitis and is not specific to any certain subtype. This rearrangement is associated with proliferation of myofibroblasts and fibroblasts in either the presence or absence of osteoid metaplasia [11].

There are no evidence-based guidelines on the treatment of ossifying fasciitis. Management of this lesion ranges from spontaneous regression to surgical excision [3,9]. Minimal research has been published on pharmacological management. An animal study demonstrated that low dose diclofenac decreased the amount of heterotopic bone formation which may be promising for use in humans [12]. Another case study showed improvement with the use of intralesional steroid injection in a patient whose lesion was deemed inappropriate for surgical excision [13].

We present a case of ossifying fasciitis in a rare anatomical location that proved difficult to diagnosis. The location in the inner thigh crease and absence of trauma are characteristics that do not fit the typical diagnostic picture of ossifying fasciitis. A limited number of similar cases have been described in the literature. In a case series by Dhillon et al. four cases of ossifying fasciitis were described in the lower extremity: three in the thigh and one in the gluteal region [7]. Cases of myositis ossificans have been reported in nearby locations including the upper thigh and hip [14,15]. Ossifying fasciitis imaging findings are undistinguishable from myositis ossificans; however ossifying fasciitis is located in the fascial plane rather than the muscle^7^ .The diagnosis of ossifying fasciitis can prove difficult due to its rarity, unusual presentation, and ability to mimic other soft tissue tumors. Histologic evaluation is often required to rule out malignant lesions [3]. Other diagnoses that must be considered in the differential include conventional nodular fasciitis, myositis ossificans, fibromatosis, desmoid tumor, foreign body granuloma, ossifying fibromyxoid tumor, and extraosseous osteosarcoma [3,8].

## Conclusion

Nontraumatic ossifying fasciitis should be considered in the differential diagnosis of soft tissue masses of the proximal lower extremities.
